# The benefits of single-subject research designs and multi-methodological approaches for neuroscience research

**DOI:** 10.3389/fnhum.2023.1190412

**Published:** 2023-10-25

**Authors:** April Becker

**Affiliations:** Department of Behavior Analysis, College of Health and Public Service, University of North Texas, Denton, TX, United States

**Keywords:** single-subject, experimental design, neuroscience, within-subject, validity

## 1. Introduction

The scientific method is neither singular nor fixed; it is an evolving, plural set of processes. It develops and improves through time as methodology rises to meet new challenges (Lakatos, 1978; Hull, 1988; Kuhn and Hacking, 2012). “It would be wrong to assume that one must stay with a research programme until it has exhausted all its heuristic power, that one must not introduce a rival programme before everybody agrees that the point of degeneration has probably been reached” (Lakatos, 1978). These insights apply not least to experimental design approaches.

For better and for worse, no experimental design comes without limitation. We must accept that the realities of the world cannot be simplistically verified against universal standard procedures; we are free instead to explore how the progressive evolution of experimental design enables new advancement. This paper proposes support for a shift of focus in the methodology of experimental research in neuroscience toward an increased utilization of single-subject experimental designs. I will highlight several supports for this suggestion. Most importantly, single-subject methods can complement group methodologies in two ways: by addressing important points of internal validity and by enabling the inductive process characteristic of quality early research. The power of these approaches has already been somewhat established by key historical neuroscience experiments. Additionally, the individuated nature of subject matter in behavioral neuroscience makes the single-subject approach particularly powerful, and single-subject phases in a research program can decrease time and resource costs in relation to scientific gains.

## 2. Complimentary research designs

Though the completely randomized group design is considered by many to be the gold standard of evidence (Meldrum, 2000), its limitations as well as ethical and logistical execution difficulties have been noted: e.g., blindness to group heterogeneity, problematic application to individual cases, and experimental weakness in the context of other often-neglected aspects of study design such as group size, randomization, and bias (Kravitz et al., 2004; Grossman and Mackenzie, 2005; Williams, 2010; Button et al., 2013). Thus, the concept of a “gold standard” results not from the uniform superiority of a method, but from an implicit valuing of its relative strengths compared to other designs, *all things being equal* (even though such things as context, randomization, group size, bias, heterogeneity, etc. are rarely equal). There is an alternative to this approach. Utilizing a wider array of methods across studies can help compensate for the limitations of each and provide flexibility in the face of unequal contexts. In a multi-methodological approach, different experimental designs can be evaluated in terms of complementarity rather than absolute strength. If one experimental design is limited in a particular way, adding another approach that is stronger in that aspect (but perhaps limited in another) can provide a more complete picture. This tactic also implicitly acknowledges that scientific rigor does not proceed only from the single study; replication, systematic replication, and convergent evidence may proceed from a progression of methods.

I suggest adding greater utilization of single-subject design to the already traditionally utilized between-subject and within-subject group designs in neuroscience to achieve this complementarity. The advantages and limitations of these designs are somewhat symmetrical. Overall, single-subject experiments carry with them more finely-focused internal validity because the same subject (together with their array of individual characteristics) serves in both the experimental and control conditions. Unlike in typical within-subject group comparisons, the repetition of comparisons in single-subject designs control for other confounding variables, rendering *n* = 1 into a true experiment. While an unreplicated single-subject experiment by itself cannot establish external validity, systematic replication of single-subject experiments over the relevant range of individual differences can. On the other hand, group designs cannot demonstrate an effect on an individual level, but within-individual group studies can characterize the generality of effects across large populations in a single properly sampled study, and may be particularly suited to analyzing combined effects of multiple variables (Kazdin, 1981). Single subject and group approaches can also be hybridized to fit a study's goals (Kazdin, 2011). In the following sections, I will describe aspects of each approach that illustrate how the addition of single-subject methodology to neuroscience could be of use. I do not mean to exhaustively describe either methodology, which would be outside the scope of this paper.

### 2.1. Group designs

Group experimental designs^1^ interrogate the effect of an independent variable (IV) by applying that variable to a group of people, other organisms, or other biological units (e.g., neurons) and usually—but not always—comparing an aggregated population measure to that of one or more control groups. These designs require data from multiple individuals (people, animals, cells, etc.). Group experiments with between-group comparisons often assign these individuals to conditions (experimental or control) randomly. Other group experiments (such as a randomized block design) assign individuals to conditions systematically to explicitly balance the groups according to particular pre-considered individual factors. In both cases, the assumption is that if alternative variables influence the dependent variable (DV), they are unlikely to do so differentially across groups. Group experiments with within-subject comparisons expose each individual to both experimental and control conditions at different times and compare the grouped measures between conditions; this approach assures that the groups are truly identical since the same individuals are included in both conditions.

Because they involve multiple individuals, some group designs can provide important information about the generality of an effect across the included population, especially in the case of within-subject group designs. Unfortunately, some often-misused aspects of group designs tend to temper this advantage. For example, restricted inclusion criteria are often necessary to produce clear results. When desired generality involves only such a restricted population (e.g., only acute stroke patients, or only layer IV glutamatergic cortical neurons), this practice carries no disadvantage. However, if the study aims to identify more widely applicable processes, stringent inclusion criteria can produce cleaner but overly conditional results, limiting external validity (Henrich et al., 2010). Further, the analysis approach taken in many group designs that narrowly examines changes in central tendency (such as the mean) of groups can limit the assessment of generality within the sampled population since averaging will wash out heterogeneity of effects. Other aspects of rigor in group designs can also affect external validity (e.g., Kravitz et al., 2004; Grossman and Mackenzie, 2005; Williams, 2010; Button et al., 2013).

Another limitation of group design logic is the practical difficulty of balancing individual differences between groups. In the case of between-group comparisons, these difficulties arise from selection bias, mortality, etc. Even well controlled studies can still produce probabilistically imbalanced groups, especially in the small sample sizes often used in neuroscience research (Button et al., 2013). Deliberately balanced groups or *post-hoc* statistical control may help, but the former introduces a potential problem with true randomization, and the latter is weaker than true experimental control. Within-subject group comparisons implement both experimental and control conditions for each individual in a group and therefore better control for individual differences, however these designs still do not experimentally establish effects within the individual since single manipulations of experimental conditions can be confounded with other changes on an individual level.

The typical focus on parameters such as the mean in the analysis of group designs can also threaten internal as well as external validity, particularly if the experimental question concerns biological or behavioral variables that are highly individually contextualized or developmentally variant.^2^ This problem extends from the fact that aggregate measures across populations do not necessarily reflect any of the underlying individuals (e.g., Williams, 2010); for example, average brain functional mapping tends not to apply to individual brains (Brett et al., 2002; Dworetsky et al., 2021; Fedorenko, 2021; Hanson, 2022). This kind of problem is particularly amplified in the study of human behavior and brain sciences, which both tend to be highly idiosyncratic. In these cases, aggregated measures can mask key heterogeneity including contradictory effects of IVs. This can complicate the application of results to individuals: an issue especially relevant in clinical research (Sidman, 1960; Williams, 2010). Relatedly, the estimation of population-based effect size provides scant information with which to estimate effects and relevance for an individual. *Post-hoc* statistical analysis may help to tease out these issues, but verification still requires new experimentation. True generality of a scientific insight requires not only that effects occur with reasonable replicability across individuals, but that a reasonable range of conditions that would alter the effect can be predicted: a difficult point to discern in group studies. Thus, while group designs carry advantages insofar as they can be used to characterize effects across a whole population in a single experiment, those advantages can be and often are subverted. Perhaps counter-intuitively, single-subject approaches can be ideal for methodically discovering the common processes that underlie diversity within a population, which have made it particularly powerful in producing generalizable results (see next section).

### 2.2. Single-subject designs

Single-subject designs compare experimental to control conditions repeatedly over time within the same individual. Like group designs with within-subject comparisons, single-subject designs can control for individual differences, which remain constant. However, single-subject designs take individual control to a new level. Since other confounding changes may coincide with a single change in the IV, single-subject designs also require multiple implementations of the same manipulation so that the comparison can be repeated within the individual, controlling for the coincidental confounds of a single condition change. Additionally, single-subject designs measure multiple data points through time within each condition before any experimental change occurs to assess pre-existing variation and trends in comparisons with the subsequent condition. Of course, a single-subject experiment without inter-individual replication has no generality—systematic replications across relevant individual characteristics and contexts are generally required to establish external validity. However, the typical group design also often requires similar replication to establish the same validity, and unlike group designs single-subject studies are also capable of rigorously interrogating even the rarest of effects.

Because single-subject experiments deal well with individual effects, they are often used in clinical and closely applied disciplines, e.g., education (Alnahdi, 2015), rehabilitation and therapy (Tankersley et al., 2006), speech and language (Byiers et al., 2012), implementation science (Miller et al., 2020), neuropsychology (Perdices and Tate, 2009), biomedicine (Janosky et al., 2009), and behavior analysis (Perone, 1991). However, the single-subject design is not limited to clinical applications or to the study of rare effects; it can also be used for the study of generalizable individual processes via systematic replication. Serial replications often enable detailed distillation of both common and uncommon relevant factors across individuals, making the approach particularly powerful for identifying generalizable processes that account for within-population diversity (although this process can be challenging even on the single-subject level; see Kazdin, 1981). Single-subject methodology has historically established some of the most generalizable findings in psychology including the principles of Pavlovian and operant conditioning (Iversen, 2013). Establishing this generalizability requires a research program rather than a single study, however since each replication (and comparisons between them) can potentially add information about important contextual variables, systematic progression toward generality can be more efficient than in one-shot group studies.

Single-subject designs are sometimes confused with within-subject group comparisons or n-of-1 case studies, neither of which usually include multiple implementations of each condition for any one individual. N-of-1 case studies sometimes make no manipulation at all or may make a single comparison (as with an embedded AB design or pre-post observation), which can at best serve as a quasi-experiment (Kazdin and Tuma, 1982). A single subject design, in contrast, will include many repeated condition changes and collect multiple data points inside each condition (as in the ABABABAB design as well as many others, see Perone, 1991). As is the case for group designs, the quality of evidence in a single-subject experiment increases with the number of instances in which the experimental condition is compared to a control condition; the more comparisons occur, the less likely it is that an alternative explanation will have tracked with the manipulation. A strong single-subject design will require a minimum of three IV implementations for the same individual (i.e., ABABAB, with multiple data points for each A and each B), and a robust effect will require many more.

Because single-subject designs implement conditions across time, they are susceptible to some important limitations including sequence, maturation, and exposure effects. The need to consider within-condition stability, serial dependence in data sets, reversibility, carryover effects, and long experimental time courses can also complicate these designs. Still, manipulations common in neuroscience research is often amenable to these challenges (Soto, 2020). Single-subject designs for phenomena that are not reversable (such as skill acquisition) can also be studied using approaches such as the within-subject multiple baseline. Multiple baselines experiments across behaviors, across cell populations, or across homotopic brain regions may be reasonable if independence can be established (Soto, 2020). A variety of single-subject methods are available that can help to address the unique strengths and limitations in single-subject methodology; the reader is encouraged to explore the variety of designs that cannot be enumerated in the scope of the current paper (Horner and Baer, 1978; Hains and Baer, 1989; Perone, 1991; Holcombe et al., 1994; Edgington, 1996; Kratochwill et al., 2010; Ward-Horner and Sturmey, 2010).

### 2.3. A note about statistical methods

Issues relating to statistical analysis are commonly erroneously conflated with group experimental design *per se*. Problems with the frequentist statistical approach commonly used in group designs has greatly impacted its efficacy; frequentist statistical methods carry limitations that have been treated thoroughly elsewhere [e.g., the generic problems with null-hypothesis statistical testing NHST (Branch, 2014), the inappropriate use of frequentist statistics contrary to their best use and design (Moen et al., 2016; Wasserstein and Lazar, 2016), and the inappropriate reliance on *p*-values (Wasserstein and Lazar, 2016)]. I do not expand on these issues in my summary of group design because such critiques need not apply to all between-group comparisons. The use and applicability of analysis techniques are separable from the experimental utility of group designs in general, which are not limited to inferential statistics. Group experiments can also be analyzed using alternative, less problematic statistical approaches such as the probability of replication statistic or P-rep (Killeen, 2015) and Bayesian approaches (Berry and Stangl, 2018). Well-considered statistical best practices for various forms of group analysis (e.g., Moen et al., 2016) can help a researcher to address limitations.

The conflation of statistical methods with group designs has also led to the misconception that single-subject designs cannot be analyzed statistically. Most scientists have less familiarity with statistical analyses appropriate for use in single-subject designs and the serially-dependent data sets that they produce. While pronounced effects uncovered in single-subject experiments can often be clearly detected using appropriate visual analysis, rigorous statistical methods applicable to single-subject designs are also available (e.g., Parker and Brossart, 2003; Scruggs and Mastropieri, 2013).

## 3. Single-subject design and the inductive process

The advantages highlighted above suggest not only compatibility between single-subject and group approaches, but a potential advantage conferred by an order of operations between methods. Early in the research process, inductive inference based on single-subject manipulations are ideal to generate likely and testable abstractions (Russell, 1962). Using single-subject approaches for this inductive phase requires fewer resources compared to fully powered group approaches and can be more rigorous than small-n group pilots. An effect can be isolated in one individual, then systematically replicated across relevant differences and contexts until it fails to replicate, at which time explanatory variables can be adjusted until replicated results are produced. The altered experiment can then be analyzed in comparison to previous experiments to form a more general understanding that can be tested in a new series of experiments. After sufficient systematic replication, hybrid and group designs can assess the extent to which inductively and contextually informed abstractions generalize across the widest relevant populations.

## 4. Precedent of within-subject methods

Although within-subject group experiments are common in human neuroscience and psychology, e.g., Greenwald (1976) and Crockett and Fehr (2014), full-fledged single-subject designs are virtually unknown in many subfields. Still, high-impact neuroscience experiments have occasionally either implicitly or deliberately implemented within-subject reversals, demonstrating the power of these approaches to advance the science. To name just a few high-impact examples, Hodgkin et al. (1952) classic work on voltage clamping utilizing the giant squid neuron involved multiple parametric IV implementations on single neurons. The discovery of circadian rhythms in humans also involved systematic single-subject experiments comparing circadian patterns at various light intensities, light-dark schedules, and control contexts, which allowed investigators to establish that outside entrainment overrode the cycle-altering effects of different light intensities (Aschoff, 1965). This fruitful precedent of single-subject-like experiments at the very foundation of historical neuroscience together with the well-established efficacy of single-subject design in other fields imply that the wider adoption of the full methodology can succeed.

## 5. Single-subject design and individuality in neuroscience

As suggested earlier in this paper, individual variation dominates the scene in behavioral and brain sciences and constitutes a basic part of the evolutionary selection processes that shaped them. In human neuroscience, individual developmental and experience-dependent variation are of particular importance. Human brains are so individuated that functional units across individuals cannot be discerned via typical anatomical landmarks, and even between-group designs often need to utilize individuated or normalized measures (Brett et al., 2002; Dworetsky et al., 2021; Fedorenko, 2021; Hanson, 2022). A shift toward including rigorous single-subject research therefore holds particular promise for the field. For example, systematically replicated individual analyses of functional brain networks and their dynamics may more easily lead to generalizable ideas about how they develop and change, and these purportedly general processes could in turn be tested across individual contexts.

## 6. Time and resource logistics

Group methodology often requires great time and resources in order to produce properly powered experiments. This can lead to problems with rigor, particularly in contexts of limited funding and publish-or-perish job demands (Bernard, 2016; Button, 2016). Especially in early stages of research, single-subject methodology enables experimenters to investigate effects more critically and rigorously for each subject, to more quickly answer and refine questions in individuals first before systematically exploring the generality of findings or the importance of context, and to do so in a cost-effective way. Thus, both cost and rigor could be served by conscientiously adding single-subject methodology to the neuroscience toolbelt.

## 7. Suggestions for neuroscience subfields that could benefit

Cognitive, behavioral, social, and developmental neuroscience each deal with individual variation in which later stages are often dependent on earlier stages and seek to identify generalizable processes that produce variant outcomes: a task for which the single-subject and multi-method approach is ideal. Neurology and clinical neuroscience also stand to benefit from a more rigorous tool for investigating clinical cases or rare phenomena. While I do not mean to suggest that the method's utility should be limited to these subfields, the potential benefit seems particularly pronounced.

## 8. Discussion

In summary, greater utilization of single-subject research in human neuroscience can complement current methods by balancing the progression toward internal and then external validity and enabling a low-cost and flexible inductive process that can strengthen subsequent between-group studies. These methods have already been incidentally utilized in important neuroscience research, and they could be an even more powerful, thorough, cost-efficient, rigorous, and deliberate ingredient of an ideal approach to studying the generalizable processes that account for the highly individuated human brain and the behavior that it enables.

## Author contributions

AB conceived of and wrote this manuscript.

## Footnotes

^1^This discussion intentionally excludes assignment to groups based on non-manipulable variables because of the qualitative difference between correlational approaches and true experimental approaches that manipulates the IV. The former carries a very different set of considerations outside the scope of this paper.

^2^If the biological process under investigation actually occurs at the population level (e.g. natural selection), the population parameter precisely applies to the question at hand. However, group comparisons are more often used to study processes that function on the individual level.
